# Integrative transcriptional characterization of cell cycle checkpoint genes promotes clinical management and precision medicine in bladder carcinoma

**DOI:** 10.3389/fonc.2022.915662

**Published:** 2022-08-11

**Authors:** Wei-Wei Shi, Jing-Zhi Guan, Ya-Ping Long, Qi Song, Qi Xiong, Bo-Yu Qin, Zhi-Qiang Ma, Yi Hu, Bo Yang

**Affiliations:** ^1^ Department of Medical Oncology, Senior Department of Oncology, The Fifth Medical Center of People’s Liberation Army (PLA) General Hospital, Beijing, China; ^2^ School of Medicine, Nankai University, Tianjin, China

**Keywords:** bladder carcinoma, cell cycle checkpoints, prognostic signature, nomogram, *FGFR3*, TME, chemotherapy, immunotherapeutic treatment response

## Abstract

**Background:**

The aberrant regulation of cell cycle is significantly correlated with cancer carcinogenesis and progression, in which cell cycle checkpoints control phase transitions, cell cycle entry, progression, and exit. However, the integrative role of cell cycle checkpoint-related genes (CRGs) in bladder carcinoma (BC) remains unknown.

**Methods:**

The transcriptomic data and clinical features of BC patients were downloaded from The Cancer Genome Atlas (TCGA), used to identify CRGs correlated with overall survival (OS) by univariate Cox regression analysis. Then, the multivariate and least absolute shrinkage and selection operator (LASSO) Cox regression analyses further developed a prognostic CRG signature, which was validated in three external datasets retrieved from Gene Expression Omnibus (GEO). The receiver operating characteristic curve (ROC) analysis was conducted for evaluating the performance of the CRG signature in prognosis prediction. RNA sequencing (RNA-Seq) was performed to explore the expression difference in the identified CRGs between tumor and normal tissue samples from 11 BC patients in the local cohort. Ultimately, genomic profiles and tumor microenvironment (TME), and the Genomics of Drug Sensitivity in Cancer (GDSC) were investigated to guide precision treatment for BC patients with different CRG features.

**Results:**

The novel constructed 23-CRG prognostic signature could stratify BC patients into high-risk and low-risk groups with significantly different outcomes (median OS: 13.64 vs. 104.65 months). Notably, 19 CRGs were the first to be identified as being associated with BC progression. In three additional validation datasets (GSE13507, GSE31684, and GSE32548), higher CRG scores all indicated inferior survival, demonstrating the robust ability of the CRG signature in prognosis prediction. Moreover, the CRG signature as an independent prognostic factor had a robust and stable risk stratification for BC patients with different histological or clinical features. Then, a CRG signature-based nomogram with a better performance in prognostic prediction [concordance index (C-index): 0.76] was established. Functional enrichment analysis revealed that collagen-containing extracellular matrix (ECM), and ECM-related and MAPK signaling pathways were significantly associated with the signature. Further analysis showed that low-risk patients were characterized by particularly distinctive prevalence of *FGFR3* (17.03% vs. 6.67%, *p* < 0.01) and *POLE* alterations (7.97% vs. 2.50%, *p* < 0.05), and enrichment of immune infiltrated cells (including CD8+ T cells, CD4+ naïve T cells, follicular helper T cells, Tregs, and myeloid dendritic cells). RNA-seq data in our local cohort supported the findings in the differentially expressed genes (DEGs) between tumor and normal tissue samples, and the difference in TME between high-risk and low-risk groups. Additionally, CRG signature score plus *FGFR3* status divided BC patients into four molecular subtypes, with distinct prognosis, TME, and transcriptomic profiling of immune checkpoint genes. Of note, CRG signature score plus *FGFR3* status could successfully distinguish BC patients who have a higher possibility of response to immunotherapy or chemotherapy drugs.

**Conclusions:**

The CRG signature is a potent prognostic model for BC patients, and in combination with *FGFR3* alterations, it had more practical capacity in the prediction of chemotherapy and immunotherapy response, helping guide clinical decision-making.

## Introduction

Cell cycle checkpoint-related genes (CRGs) play pivotal roles in cell cycle progression (CCP), ensuring and control regulating cell cycle events (1). Generally, in eukaryotic cells, the mitotic cell cycle is composed of two stages, the interphase (G1, S, and G2) and the mitotic (M) phase. The gap phases of G1-to-S (2), S-to-G2 (3), and G2-to-M (4), likely as decision windows, can determine cell cycle entry and progression. In cancer cells, some CRGs preventing DNA damage are usually compromised, contributing to genetic alterations and genomic instability (5), but those CRGs involved in DNA replication stress are scarcely altered to endure the replication stress (6). On the other hand, cancer cells could potentiate DNA replication stress through transcriptional regulation of CRGs (7). Overall, CRGs and CRG-related signaling pathways play a key role in regulating the phase transitions, CCP, and cell cycle entry and exit in cancer cells (8).

In previous studies, it has been revealed that aberrantly expressed CRGs might be an essential prerequisite for cells to become cancerous, leading to tumor development and progression. For instance, CRG ataxia telangiectasia mutated (*ATM*) plays a core role in responding to DNA damage and stimulating DNA repair signaling pathways, and its absence is highly prone to giving rise to carcinogenesis (9). Of note, its downregulation or inactivation is associated with the highly accumulated genomic aberrations, which is one of the hallmarks of cancer (10). Ataxia telangiectasia and Rad3-related (*ATR*), another CRG encoding protein kinase that regulates DNA damage response (DDR), is correlated with the polarization of M2 tumor-associated macrophages, lymph node metastasis, and poor prognosis of patients with nasopharyngeal carcinoma (11). The abnormal expression of CRGs has been found to be associated with the development and progression of multiple kinds of cancers, including melanoma (12), lung cancer (13), colorectal cancer (14), and hepatocellular carcinoma (15). A growing body of evidence has found that the dysregulation of CRGs could render cells to be cancerous and promote cancer cell proliferation, but most studies only disclose the role of a single CRG in cell cycle, tumor carcinogenesis, and progression. Currently, the role of integrated CRGs representing checkpoint mechanisms in the regulation of cell cycle in tumor carcinogenesis and progression remains to be fully delineated.

Previous studies have already expounded that dysregulation of CRGs is correlated with increased genomic instability and malignant progression in bladder carcinoma (BC) patients (16–18), indicating that there is a great potential of CRGs to become prognostic or targeted biomarkers for BC patients. BC generally presents as non-muscle-invasive bladder cancer (NMIBC), muscle-invasive bladder cancer (MIBC), or metastatic BC, of which the MIBC subtype has a relatively worse prognosis and poor treatment responses (19). Moreover, BC is a molecularly heterogeneous cancer with divergent clinical outcomes (20). The heterogeneity of tumor is always a huge challenge for cancer management and could reduce the efficacy of molecularly targeted therapies; therefore, the dissection of molecular signatures is urgently needed. Based on transcriptomic profiling of CRGs, a novel signature was constructed in the present study with good performance in prognosis prediction and that could function as a biomarker for treatment response. Overall, this study was of guiding significance in the clinical management of BC patients and promoting precision treatment.

## Materials and methods

### Data acquisition

In the current study, RNA-seq data (TPM: transcripts per million) and related clinical features of BC patients involved in the TCGA-BC cohort were collected (https://www.cbioportal.org/) as the training cohort (BC samples with no available RNA-seq data or survival information were excluded in subsequent analyses). The transcriptional profiles together with clinical features of GSE13507 (including 62 MIBC and 103 NMIBC patients), GSE31684 (including 79 MIBC and 14 NMIBC patients), and GSE32548 (including 131 BC patients; the muscle-invasive status was uncertain) were downloaded, as three independent validation groups, from GEO (https://www.ncbi.nlm.nih.gov/geo/). The tumor and normal tissue samples from the TCGA, GSE133624, GSE188715, and GSE13507 cohorts were retrieved to conduct the differentially expressed gene (DEG) analysis. The RNA-seq data were normalized by Log_2_(x+0.001). Cell cycle checkpoint gene sets were obtained from the database of Gene Ontology (GO), Biological Process (http://geneontology.org/), and Reactome (https://reactome.org/). The term “cell cycle checkpoint” was used for the acquisition of CRGs. After merging, a total of 464 CRGs were included in the union set, which were then selected for the further processes of establishing a prognostic signature.

### Identification of the prognostic CRG signature

In order to investigate whether transcriptomic characterization was associated with prognosis of BC patients, the unsupervised hierarchical clustering analysis was conducted by using the package “Fastcluster”, dividing BC patients into different clusters. The principal component analysis (PCA) was performed *via* the “ggbiplot” package, further revealing the distinction between clusters. The Kaplan–Meier curve analysis showed the overall survival (OS) of patients between clusters. Subsequently, the univariate Cox proportional hazard regression analysis, by using the packages “rms” and “survival”, was conducted to classify the relationship of OS and the expression of each CRG in the TCGA-BC cohort. The least absolute shrinkage and selection operator (LASSO) and multivariate Cox proportional hazard regression analyses were used to construct a prognostic CRG signature *via* the “glmnet” package. Subsequently, a risk formula was established, and the CRG score was generated for each BC patient with the following formula: CRG score = expression value of gene 1 × C_1_ + expression value of gene 2 × C_2_ + … + expression value of gene x × C_x_, where C_x_ is the coefficient of gene x. The optimal CRG score was adapted as the cutoff value by using the package “maxstat” to divide BC patients into high-risk and low-risk groups, while the same method was utilized in the validation groups. The value of area under the receiver operating characteristic (ROC) curve (AUC) revealed the prognostic performance of specificity and sensitivity. All analyses were also applied in the validation groups.

### RNA sequencing in the local BC cohort

Tumor and matched normal tissues were collected from 11 MIBC patients in our local cohort to perform RNA-seq. This study was approved by the ethics committee of the Chinese PLA General Hospital (S2019–302-01), and all enrolled patients have signed the informed consent. The total RNA of each sample was collected using a FastPure^®^ Cell/Tissue Total RNA Isolation Kit V2 (Vazyme, Jiangsu, China), and its concentration and RNA Integrity Number (RIN) were determined by using Qubit (Thermo Fisher Scientific, MA, United States) and an Agilent 2100 bioanalyzer (Agilent Technologies, CA, United States), respectively. One sample of normal tissue failed quality control and then was discarded. Library construction was conducted using the NEBNext^®^ Ultra™ RNA Library Prep Kit for Illumina^®^ Kit (NEB, MA, United States) and finally sequenced on the Illumina Novaseq-6000 system (Illumina, MA, United States).

### Construction of the predictive nomogram

The univariate and multivariate Cox proportional hazard regression analyses, by using the packages “rms” and “survival”, were conducted to explore whether the CRG signature was an independent prognostic factor. Furthermore, the OS-related clinical features were selected for the construction of a CRG signature-based nomogram. The value of AUC was used to evaluate the performance of a novel constructed nomogram in prognosis prediction, and the calibration plots were built to perform the consistency between actual OS and predicted OS by using the package “rms”.

### Functional enrichment analysis

The Pearson correlation analysis was conducted to screen out the CRG score-associated genes by the cutoff criteria of |Pearson Correlation Coefficient| > 0.4 and *p* < 0.05, and the heatmap showing the expression of the identified genes was drawn by using the package “pheatmap”. The Gene Ontology (GO) and Kyoto Encyclopedia of Genes and Genomes (KEGG) pathway enrichment analyses were conducted based on the expression of CRG score-associated genes.

### Tumor microenvironment analysis

The stromal score and immune score could predict the infiltration levels of stromal and immune cells, respectively. Moreover, the ESTIMATE (Estimation of STromal and Immune cells in MAlignant Tumor tissues using Expression data) algorithm was used to determine the tumor purity in tumor tissues (21). The CIBERSORT (Cell type Identification By Estimating Relative Subsets Of known RNA Transcripts) algorithm was a widely used method to characterize the tumor-infiltrated lymphocytes (TILs) inside tumor tissues based on the gene expression profile (22); thus, the abundance of TILs between different groups was compared by the evaluation of CIBERSORT. The T-cell dysfunction score, exclusion score, and Tumor Immune Dysfunction and Exclusion (TIDE) score were analyzed to estimate the tumor immune escape (23). Furthermore, the expression levels of immune checkpoint genes, such as *PD-1*, *PD-L1*, *CTLA-4*, *TIM-3*, and *LAG3* (24), were investigated to explore the potential immune therapies for different BC patients.

### Evaluation of therapeutic treatment responses

The data were downloaded from the Genomics of Drug Sensitivity in Cancer (GDSC, https://www.cancerrxgene.org/), a publicly available pharmacogenomic database, to predict the treatment response of chemotherapy for BC patients. The measuring parameter of half-maximal inhibitory concentration (IC_50_) was used to estimate the chemotherapeutic treatment response and chemo-drug sensitivity between different groups *via* the “pRRophetic” package. In addition, the clinical information and transcriptomic data of 348 urothelial cancer (UC) patients involved in the IMvigor210 cohort receiving anti-PD-L1 therapy (Atezolizumab) were downloaded from the following website (25): http://research-pub.gene.com/IMvigor210CoreBiologies/. The criteria of treatment response were defined as previously described: CR: complete response, PR: partial response, SD: stable disease, PD: progressive disease. In addition, the GSE176307 dataset (26), including 86 UC patients with wild-type *FGFR3* and 17 patients with altered *FGFR3* receiving anti-PD-1 or anti-PD-L1 treatment, was further employed to explore the response prediction ability of the CRG signature for BC patients with different molecular subtypes by immunotherapeutic treatment.

### Statistical analysis

The statistical data were analyzed by the Kruskal–Wallis (K-W) test, Mann–Whitney *U* test, Chi-square test, and Fisher’s exact test in R studio. Cluster analysis was performed by the unsupervised hierarchical clustering. The univariate and multivariate Cox proportional hazards regression models were employed to assess the hazard ratio of the signature and clinical features. The Kaplan–Meier curve analysis along with log-rank test was conducted to evaluate the clinical outcomes of BC patients. The statistically significant difference was determined by “*p* < 0.05”.

## Results

### The expression profiling of CRGs correlated with OS of BC patients

**Figure 1:**
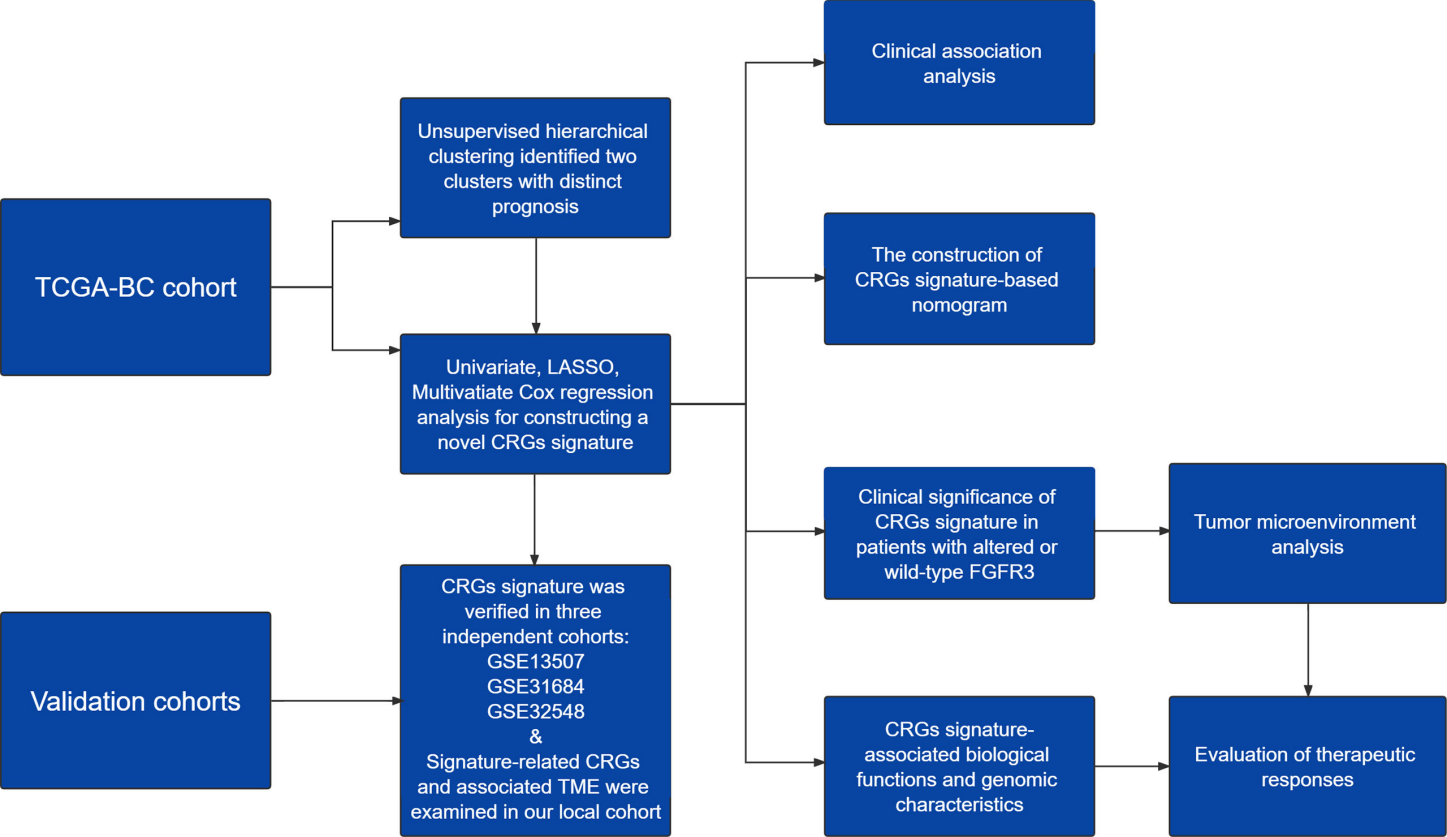
** **A work flowchart of constructing a novel cell cycle checkpoint gene (CRG) signature, with predictive abilities for prognosis and treatment response in BC.

**Figure 2:**
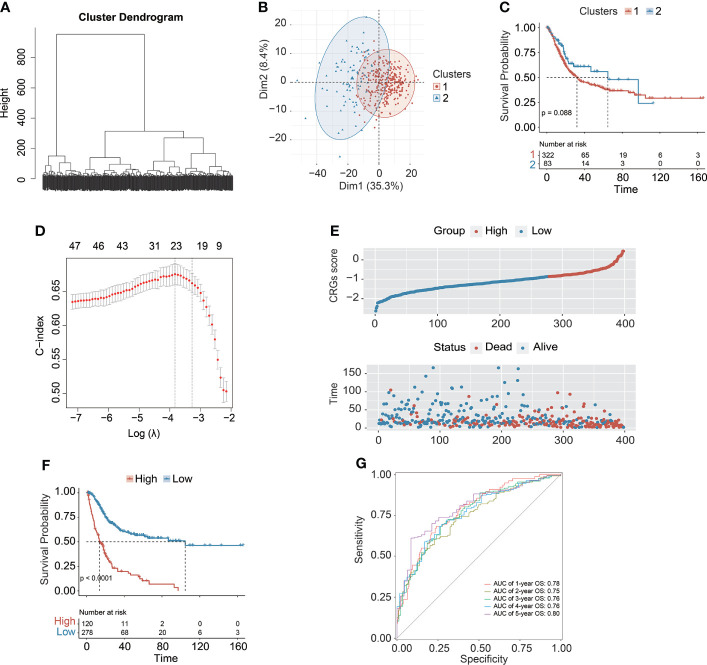
The transcriptomic profiling of cell cycle checkpoint-related genes (CRGs) was correlated with overall survival (OS) of BC patients from the TCGA-BC cohort. **(A)** The unsupervised hierarchical clustering of BC patients based on the transcriptional characterization of CRGs. **(B)** The principal component analysis (PCA) showed the differentiation between clusters 1 and 2. **(C)** Kaplan–Meier curve analysis to compare OS between clusters 1 and 2. **(D)** The least absolute shrinkage and selection operator (LASSO) Cox regression analysis for construction of the prognostic CRG signature. **(E)** The scatterplot demonstrated the distribution of CRG scores corresponding to survival status of BC patients in the training cohort. **(F)** Kaplan–Meier curve analysis to compare OS between high-risk and low-risk groups. **(G)** The receiver operating characteristic curve (ROC) analysis for evaluating the ability of the CRG signature in prognosis prediction.

The design of this study was exhibited in a work flowchart (**Figure 1**). Initially, by way of the unsupervised hierarchical clustering analysis, BC patients from the TCGA cohort were divided into two clusters (**Figure 2A**), which could be separated in the Dim2 axis (**Figure 2B**), indicating that the expression profiling of CRGs between two clusters was noticeably distinct. Of note, BC patients in cluster 1 had worse OS (median OS: 32.02 vs. 64.80 months, *p* = 0.088, **Figure 2C**). The above results suggested that the expression profiling of CRGs potentially affected the clinical outcomes in BC.

### Identification and validation of the prognostic CRG signature

Subsequently, a total of 398 BC patients (**Table 1**) were selected into the univariate Cox proportional hazard regression analysis to investigate the relationship between OS and the expression of 464 CRGs (**Supplementary Table 1**), and eventually, expression levels of 52 CRGs were significantly correlated with the OS of BC patients (**Supplementary Table 2**). Subsequently, the LASSO and multivariate Cox proportional hazard regression analyses determined a novel prognostic CRG signature, consisting of 23 CRGs (**Figure 2D**, **Table 2**). The scatterplot demonstrated the distribution of CRG score and the corresponding survival status of BC patients in the training group, in which BC patients were divided into high-risk and low-risk groups (**Figure 2E**). It was identified that BC patients in the high-risk group had a significantly worse OS than those in the low-risk group (median OS: 13.64 vs. 104.65 months, *p* < 0.0001, **Figure 2F**). The AUC values of the CRG signature for predicting OS at 1, 2, 3, 4, and 5 years were 0.78, 0.75, 0.76, 0.76, and 0.80, respectively (**Figure 2G**), indicating good specificity and sensitivity of the CRG signature in predicting the prognosis of BC patients.

**Table 1:** Clinical features of 398 involved BC patients from the TCGA cohort.

Clinical Feature		Number (%)
**Total**		398 (100%)
**Gender**		
	Male	293 (73.62%)
	Female	105 (26.38%)
**Age**	Median (range)	68.5 (34-90)
**Histological subtypes**		
	MIBC	398 (100%)
**Histological grading**		
	High	377 (94.72%)
	Low	18 (4.52%)
**T stage**		
	T0	1 (0.25%)
	T1	3 (0.75%)
	T2	114 (28.64%)
	T3	190 (47.74%)
	T4	58 (14.57%)
**N stage**		
	N0	230 (57.79%)
	N1	46 (11.56%)
	N2	74 (18.59%)
	N3	7 (1.76%)
**M stage**		
	M0	189 (47.49%)
	M1	10 (2.51%)
**Clinical stage**		
	I	2 (0.50%)
	II	125 (31.41%)
	III	138 (34.67%)
	IV	131 (32.91%)

BC, bladder carcinoma; TCGA, The Cancer Genome Atlas; MIBC, muscle-invasive bladder carcinoma; NMIBC, non-muscle-invasive bladder carcinoma.

**Table 2:** The identified 23 genes involved in the prognostic CRG signature.

Gene Name	HR	95% CI	Coefficient	*p*-value
*MMAB*	1.3228	1.0520–1.6632	0.1739	0.0167
*NDEL1*	1.3404	1.0641–1.6884	0.1145	0.0128
*SLC25A15*	1.2738	1.0826–1.4989	0.0077	0.0035
*PPP2CB*	1.3489	1.0809–1.6834	0.0465	0.0081
*PSMA7*	1.3728	1.0652–1.7692	0.0210	0.0144
*PSMB5*	1.6366	1.2327–2.1729	0.0355	0.0007
*REXO2*	1.4077	1.1338–1.7479	0.0097	0.0020
*ARID3A*	1.1219	1.0233–1.2299	0.0387	0.0142
*CUL4A*	1.2793	1.0154–1.6119	0.0648	0.0367
*FBXO31*	1.3312	1.0623–1.6683	0.0579	0.0130
*PLK2*	1.1412	1.0325–1.2612	0.0086	0.0097
*PRPF19*	1.5588	1.1636–2.0883	0.4539	0.0029
*RGCC*	1.2245	1.0580–1.4172	0.0640	0.0066
*TIPIN*	1.3456	1.0983–1.6486	0.0560	0.0042
*ANAPC4*	0.6693	0.5173–0.8660	−0.1681	0.0023
*B9D2*	0.8078	0.6573–0.9929	−0.0190	0.0426
*PSMB10*	0.7598	0.6442–0.8962	−0.1493	0.0011
*PSMB8*	0.8695	0.7724–0.9788	−0.0427	0.0207
*RAD9A*	0.6603	0.5322–0.8191	−0.1605	0.0002
*CHMP4C*	0.7979	0.7107–0.8959	−0.1641	0.0001
*DDX39B*	0.6946	0.5118–0.9427	−0.4224	0.0194
*FBXO6*	0.7865	0.6592–0.9382	−0.0840	0.0076
*THOC1*	0.7686	0.6075–0.9724	−0.0270	0.0283

CRGs, checkpoint-related genes; HR, hazard ratio; CI, confidence interval.

**Figure 3:**
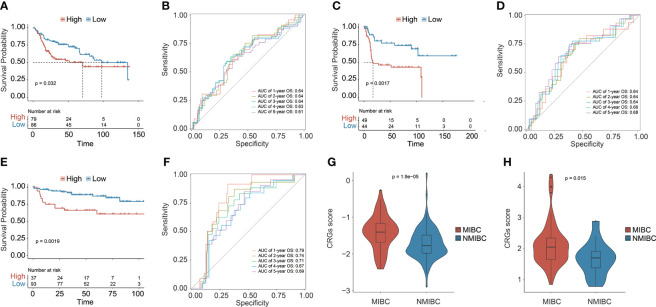
The prognostic CRG signature was validated in three independent datasets: GSE13507, GSE31684, and GSE32548. Kaplan–Meier curve analysis to compare OS between high-risk and low-risk groups in GSE13507 **(A)**, GSE31684 **(C)**, and GSE32548 **(E)**. The receiver operating characteristic curve (ROC) analysis for validating the ability of CRG signature in prognosis prediction in GSE13507 **(B)**, GSE31684 **(D)**, and GSE32548 **(F)**. Comparison analysis of the CRG scores between MIBC and NMIBC patients in GSE13507 **(G)** and GSE31684 **(H)**.

Three additional independent cohorts, namely, GSE13507, GSE31684, and GSE32548, were further employed to validate the predictive ability of the prognostic CRG signature (**Figures 3A–F**). In each validation cohort, the CRG signature had good risk stratification, and a higher CRG score indicated a significantly shorter OS (median OS: 70.73 vs. 98.00 months in GSE13507, *p* = 0.0320; 17.02 months vs. not reached in GSE31684, *p* = 0.0017; not reached vs. not reached in GSE32548, *p* = 0.0019, **Figures 3A, C, E**). The AUC values for predicting OS at 1, 2, 3, 4, and 5 years were 0.64, 0.64, 0.64, 0.63, and 0.61 in GSE13507; 0.64, 0.64, 0.64, 0.66, and 0.68 in GSE31684; 0.79, 0.74, 0.71, 0.67, and 0.69 in GSE32548, respectively (**Figures 3B, D, F**). Additionally, MIBC patients had significantly higher CRG scores compared with the NMIBC patients in both GSE13507 and GSE31684 datasets (*p* < 0.05, **Figures 3G, H**), suggesting the potential capability of the CRG signature in differentiating NMIBC and MIBC.

### Clinical association of the prognostic CRG signature

**Figure 4:**
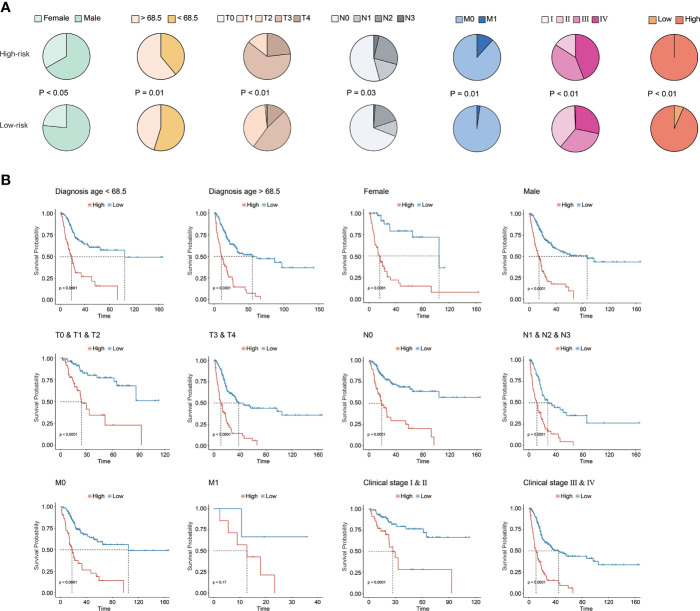
Clinical association of the CRG signature. **(A)** The association analysis between the CRG signature and clinical features in the TCGA-BC cohort. **(B)** The prognostic CRG signature predicted prognosis for BC patients with different clinical features in the TCGA-BC cohort.

Underlying the CRG signature, a significantly higher proportion of female patients as well as patients diagnosed at age over 68.5 years old were observed in the high-risk group (*p* < 0.05, **Figure 4A**). Moreover, there was also a significantly increased proportion of BC patients with advanced T (tumor stage), N (lymph node status), M (metastasis), and clinical stages (*p* < 0.05) in the high-risk group. Overall, it was demonstrated that a higher CRG score indicated advanced T, N, M, and/or clinical stages. In addition, stratification by age, gender, TNM, or clinical stages in the TCGA cohort further revealed that the CRG signature had a good predictive prognosis ability, and the higher CRG score was invariably correlated with worse survival (**Figure 4B**).

### Construction of a nomogram based on the prognostic CRG signature

**Figure 5:**
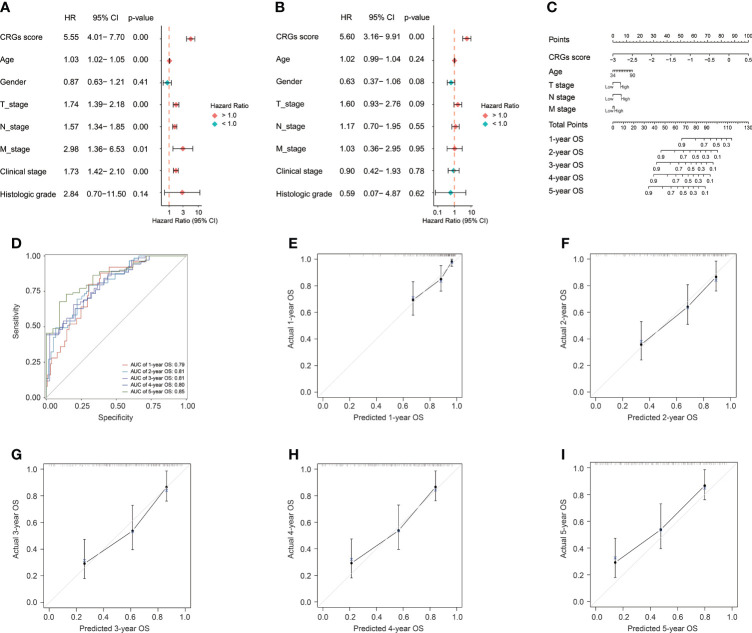
The construction of the CRG signature-based prognostic nomogram. The univariate **(A)** and multivariate **(B)** Cox regression analyses for the CRG signature and clinical features. **(C)** The constructed nomogram for the survival prediction of BC patients. **(D)** The receiver operating characteristic curve (ROC) analysis for evaluating the ability of novel constructed nomogram in prognosis prediction. The calibration curve analysis exhibited the consistency between actual OS and predicted OS by the nomogram at 1 **(E)**, 2 **(F)**, 3 **(G)**, 4 **(H)**, and 5 years **(I)**.

Apparently, the CRG signature was the most robust risk factor, by comparison with classical clinical features (**Figure 5A**), and it was the only independent prognostic factor for BC patients (*p* < 0.01, **Figure 5B**). Subsequently, the CRG signature-based prognostic nomogram was constructed in combination with several clinical parameters together, including diagnosis age, and T, N, and M stage (**Figure 5C**). The C-index of the novel constructed prognostic nomogram was 0.76, with 95% confidence interval ranging from 0.70 to 0.81. In addition, the AUC values of the nomogram for predicting OS at 1, 2, 3, 4, and 5 years were 0.79, 0.81, 0.81, 0.80, and 0.85, respectively (**Figure 5D**), and the calibration plots exhibited good consistency between actual OS and predicted OS by the nomogram (**Figures 5E–I**).

### Exploration of the CRG signature-related biological functions

**Figure 6:**
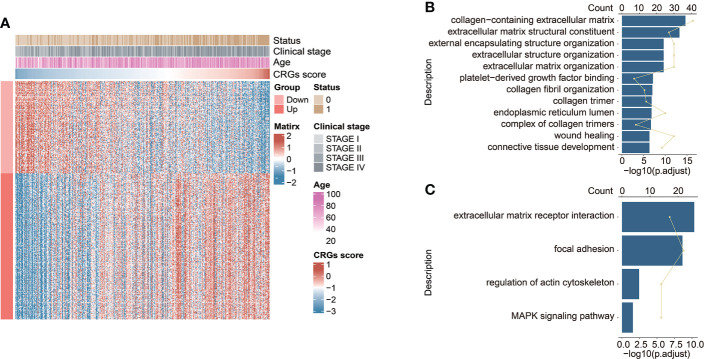
The functional enrichment analysis underlying the CRG signature. **(A)** The heatmap exhibited the expression levels of CRG signature-associated genes positively and negatively correlated with CRG score (|Pearson Correlation Coefficient| > 0.4, *p* < 0.05). The GO enrichment analysis **(B)** and KEGG pathway enrichment analysis **(C)** of the identified CRG signature-associated genes.

The highly CRG score-associated genes were defined with the criteria of |Pearson Correlation Coefficient| > 0.4 and *p* < 0.05, and a total of 252 and 159 genes were found to be positively or negatively correlated with CRG score, respectively (**Figure 6A**). The extracellular matrix (ECM)-related biological functions, focal adhesion, regulation of actin cytoskeleton, and MAPK signaling pathway were mainly enriched (**Figures 6B, C**).

### Genomic characteristics and TME underlying the CRG signature

**Figure 7:**
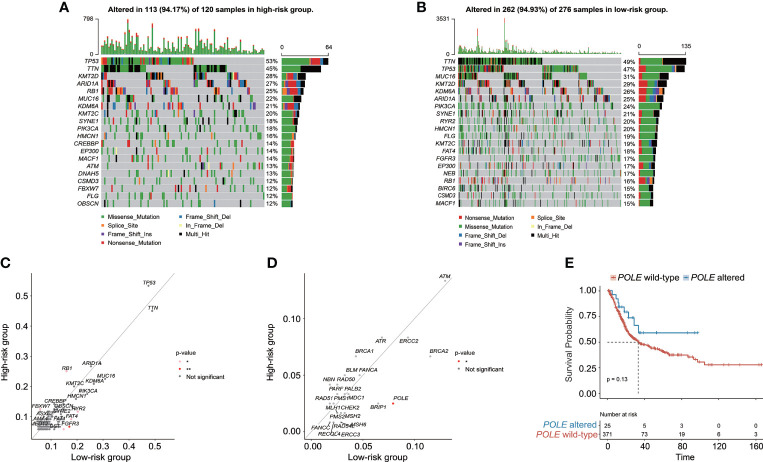
The characteristics of genomic alterations between high-risk and low-risk patients from the TCGA-BC cohort. The oncoprint plots exhibited the genomic alteration profile of the high-risk group **(A)** and low-risk group **(B)**. **(C)** The genomic alteration enrichment analysis demonstrated the prevalently altered genes between high-risk and low-risk groups. **(D)** The alteration enrichment analysis of DNA damage response (DDR) genes between high-risk and low-risk groups. **(E)** Kaplan–Meier curve analysis for BC patients with or without *POLE* alterations.

The genomic alteration profiles, respectively in high-risk and low-risk groups, showed that the most prevalently altered genes were distinct (**Figures 7A, B**). Through statistical analysis, among the prevalently altered genes between high-risk and low-risk groups (the altered genes with prevalence ≤ 5.00% in both high-risk and low-risk groups were excluded), there was a total of 46 altered genes with higher alteration frequencies enriched in the low-risk group, including *RYR2* (frequency: 20.29% vs. 11.67%, *p* < 0.05), *FAT4* (frequency: 17.75% vs. 9.17%, *p* < 0.05), and *FGFR3* (frequency: 17.03% vs. 6.67%, *p* < 0.01), whereas the high-risk group had a significantly higher prevalence of only 5 altered genes, namely, *RB1* (25.00% vs. 15.94%, *p* < 0.05), *FBXW7* (12.50% vs. 5.80%, *p* < 0.05), *NFE2L2* (10.83% vs. 3.99%, *p* < 0.05), *ASAP2* (5.83% vs. 1.45%, *p* < 0.05), and *PCSK6* (5.83% vs. 1.09%, *p* < 0.05) (**Figure 7C**). Notably, only one DDR-related gene *POLE* was found with higher alteration frequency in the low-risk group (frequency: 7.97% vs. 2.50%, *p* < 0.05, **Figure 7D**). Of note, BC patients with altered *POLE* had a trend of better clinical outcomes (median OS: 33.14 months vs. not reached, *p* = 0.13, **Figure 7E**).

**Figure 8:**
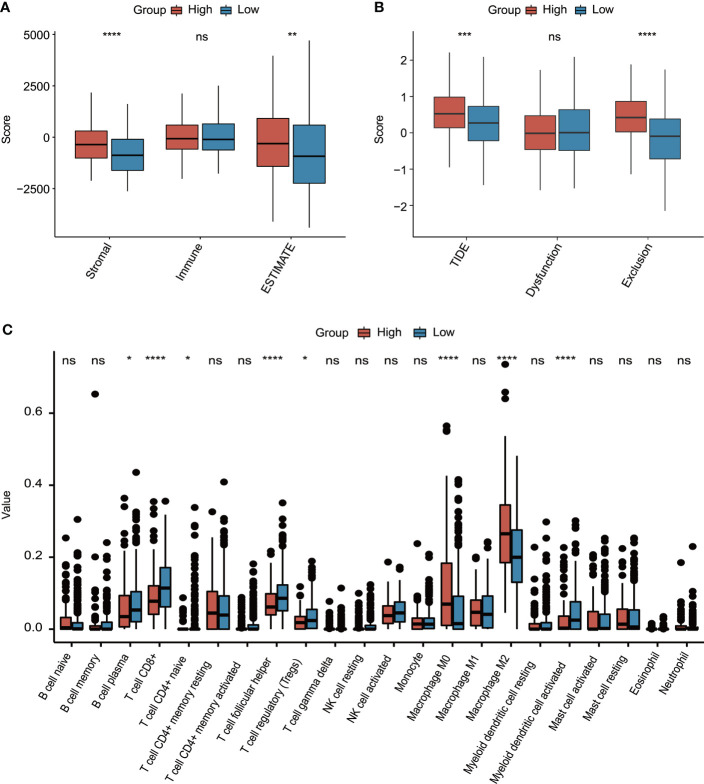
The evaluation of tumor environment between high-risk and low-risk groups. **(A)** Comparison of stromal, immune, and ESTIMATE scores between high-risk and low-risk groups. **(B)** Comparison of T-cell dysfunction, exclusion, and TIDE scores between high-risk and low-risk groups. **(C)** The evaluation of 22 tumor-infiltrated lymphocytes between high-risk and low-risk groups. *p < 0.05, **p < 0.01, ***p < 0.001, ****p < 0.0001, ns represented “not significant”.

Subsequently, the molecular characterization of TME was presented, demonstrating that the high-risk group had a significantly increased stromal score and ESTIMATE score (**Figure 8A**). Moreover, the high-risk group also had a significantly higher T-cell exclusion score and TIDE score (**Figure 8B**). Additionally, more plasma B cells, CD8+ T cells, CD4+ naïve T cells, follicular helper T cells, Tregs, and activated myeloid dendritic cells were significantly more enriched in the low-risk group, whereas the high-risk group only had significantly higher infiltration levels of macrophage M0 and M2 (**Figure 8C**).

### Exploration of CRG signature or related genes in the local BC cohort

**Figure 9:**
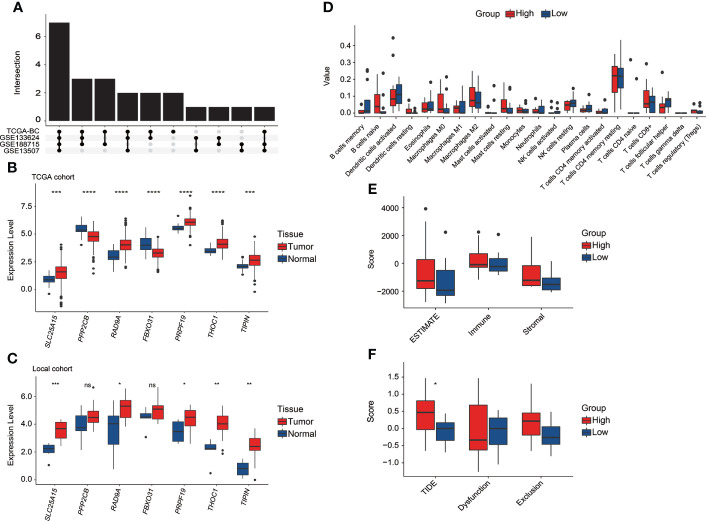
The exploration of the CRG signature and related genes in the local BC cohort. **(A)** Overlapping number of differentially expressed CRGs between tumor and normal tissue samples across the TCGA, GSE133624, GSE188715, and GSE13507 cohorts. **(B)** The comparison of seven CRG signature-related genes’ expression between tumor and normal tissue samples in the TCGA cohort. **(C)** The comparison of seven CRG signature-related genes’ expression between tumor and normal tissue samples in our local cohort. **(D)** The evaluation of 22 tumor-infiltrated lymphocytes between high-risk and low-risk groups in our local cohort. **(E)** Comparison of stromal, immune, and ESTIMATE scores between high-risk and low-risk groups in our local cohort. **(F)** Comparison of T-cell dysfunction, exclusion, and TIDE scores between high-risk and low-risk groups in our local cohort. *p < 0.05, **p < 0.01, ***p < 0.001, ****p < 0.0001, ns represented “not significant”.

It was found that the DEGs between tumor and normal tissue samples significantly varied in public datasets and our local cohort, and remarkably, only 7 of the 23 discovered CRGs exhibited significant differences between tumor and normal tissue samples across the TCGA, GSE133624, GSE188715, and GSE13507 cohorts (**Figure 9A**). Among which, *SLC25A15*, *RAD9A*, *PRF19*, *THOC1*, and *TIPIN* were significantly overexpressed in tumor tissues in both the TCGA (**Figure 9B**) and our local cohorts (**Figure 9C**). *PPP2CB* and *FBXO31* were upregulated in normal tissues in the TCGA cohort, but our local samples did not differ significantly. Limited by the sample size, there was no statistically significant difference in TILs between high-risk and low-risk groups in our local cohort, whereas, a consistent trend of more CD8+ T cells, follicular helper T cells, Tregs, and activated dendritic cells were presented in the low-risk group and relatively more macrophage M0 and M2 were presented in the high-risk group in our local cohort (**Figure 9D**). In our local cohort, the ESTIMATE score was found to be relatively higher but not statistically significant in the high-risk group (**Figure 9E**), but notably, high-risk individuals in our local BC cohort had significantly higher TIDE scores (**Figure 9F**).

### Clinical significance of CRG signature in BC patients with altered *FGFR3*

**Figure 10:**
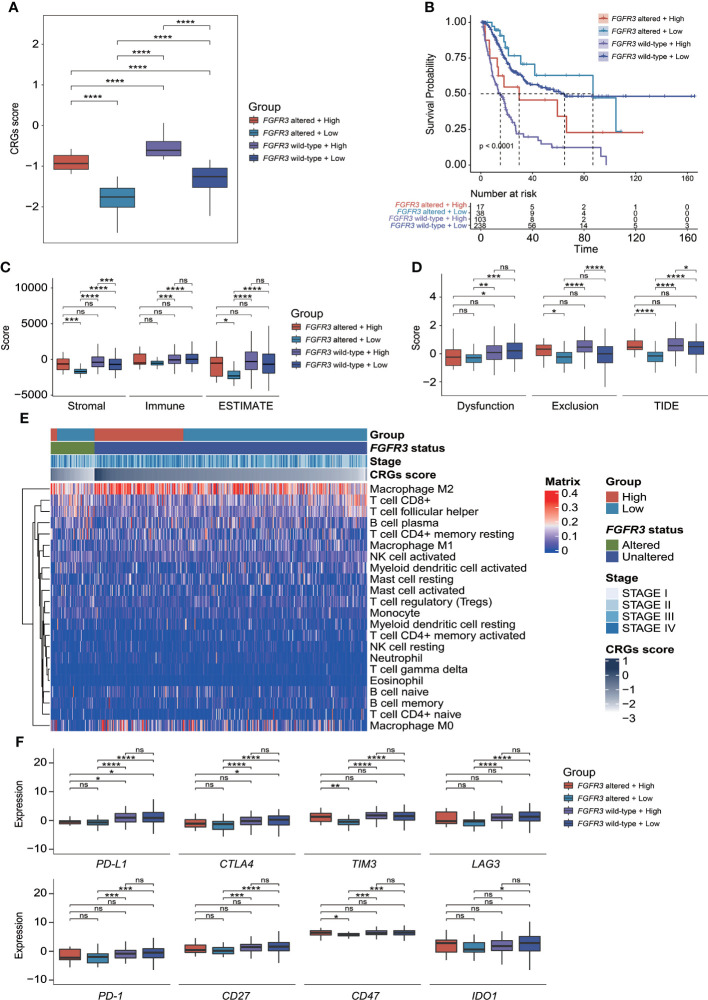
The evaluation of tumor microenvironment and immune checkpoint genes’ expression for patients stratified by CRG signature plus *FGFR3* status. **(A)** Comparison of CRG score between four molecular subgroups. **(B)** Comparison of survival *via* the Kaplan–Meier curve analysis between four molecular subgroups. **(C)** Comparison of stromal, immune, and ESTIMATE scores between four molecular subgroups. **(D)** Comparison of T-cell dysfunction, exclusion, and TIDE scores between four molecular subgroups. **(E)** The heatmap exhibited infiltrated levels of 22 tumor-infiltrated lymphocytes between four molecular subgroups. **(F)** The comparison of immune checkpoint genes’ expression levels between four molecular subgroups. *p < 0.05, **p < 0.01, ***p < 0.001, ****p < 0.0001, ns represented “not significant”.

Regarding the CRG score and *FGFR3* alteration status, BC patients were further divided into four different risk groups. It could be apparently seen that high-risk patients with wild-type *FGFR3* had the highest CRG scores (*p* < 0.0001, **Figure 10A**). Moreover, patients with wild-type *FGFR3* had significantly higher CRG scores than those with *FGFR3* alterations (*FGFR3*mt-high vs. *FGFR3*wt-high and *FGFR3*mt-low vs. *FGFR3*wt-low, *p* < 0.0001). Expectedly, high-risk patients with wild-type *FGFR3* had the significantly shortest OS (**Figure 10B**), further demonstrating the robust ability of CRG signature in prognosis prediction.

### Immunity of BC patients with altered *FGFR3* underlying the CRG signature

Compared to other groups, low-risk patients with altered *FGFR3* had the significantly lowest stromal score, immune score, ESTIMATE score, and TIDE score (**Figures 10C, D**), as well as a relatively higher enrichment of plasma B cells, activated NK cells, CD8+ T cells, and follicular helper T cells (**Figure 10E**). However, among the other three groups, there was no statistically significant difference in ESTIMATE and TIDE scores (**Figures 10C, D**), except that low-risk patients with wild-type *FGFR3* had a significantly lower TIDE score than high-risk patients with wild-type *FGFR3* (*p* < 0.05, **Figure 10D**). Remarkably, the distribution of TILs in high-risk patients with altered *FGFR3* was similar to that of BC patients with wild-type *FGFR3*, regardless of whether they had a high or low CRG score (**Figure 10E**). Interestingly, low-risk patients with altered *FGFR3* had a lower expression of immune checkpoint genes, including *PD-L1*, *CTLA4*, *TIM3*, LAG3, *PD-1*, *CD27*, *CD47*, and *IDO1* (*p* < 0.05); on the contrary, high-risk patients with altered *FGFR3* had a higher expression of *TIM3* and *CD47* (*p* < 0.05), whereas the expression level of *PD-L1* in BC patients with altered *FGFR3* was quite low (**Figure 10F**).

### Estimate of chemotherapeutic treatment response

**Figure 11:**
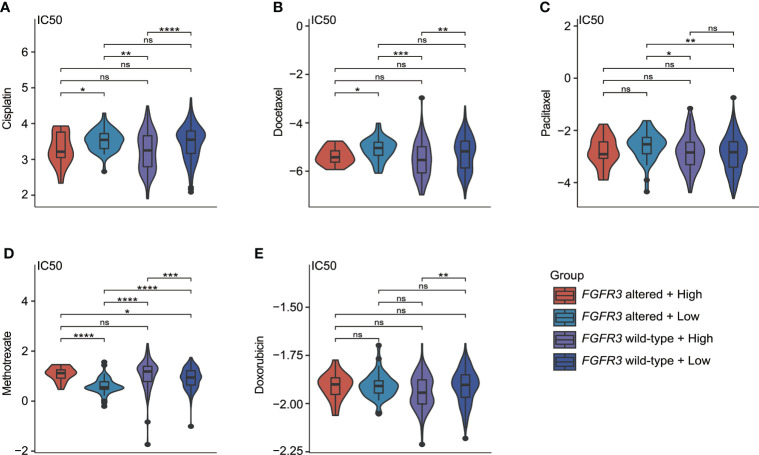
The estimate of chemotherapy treatment response *via* the GDSC database. The violin plots exhibited the IC_50_ value representing the response of chemotherapeutic treatment by cisplatin **(A)**, docetaxel **(B)**, paclitaxel **(C)**, methotrexate **(D)**, and doxorubicin **(E)** between four different molecular subgroups. *p < 0.05, **p < 0.01, ***p < 0.001, ****p < 0.0001, ns represented “not significant”.

As investigated for commonly used chemotherapeutic drugs for BC patients (including cisplatin, docetaxel, paclitaxel, methotrexate, and doxorubicin), it was found that IC_50_ values for the response prediction of chemotherapeutic treatment by cisplatin, docetaxel, paclitaxel, methotrexate, or doxorubicin significantly differed between patients with distinctive molecular subtypes (**Figures 11A–E**). Regardless of BC patients (whether *FGFR3* was altered or not), the higher CRG score indicated the relatively higher sensitivity to cisplatin and docetaxel (**Figures 11A, B**). Moreover, compared to low-risk BC patients with altered *FGFR3*, those with wild-type *FGFR3* seemed to be more sensitive to paclitaxel (**Figure 11C**). Meanwhile, low-risk patients with altered *FGFR3* had the highest sensitivity to methotrexate (**Figure 11D**). For patients with wild-type *FGFR3*, those in the high-risk group were more sensitive to doxorubicin (**Figure 11E**).

### Prediction of immunotherapeutic treatment response

**Figure 12:**
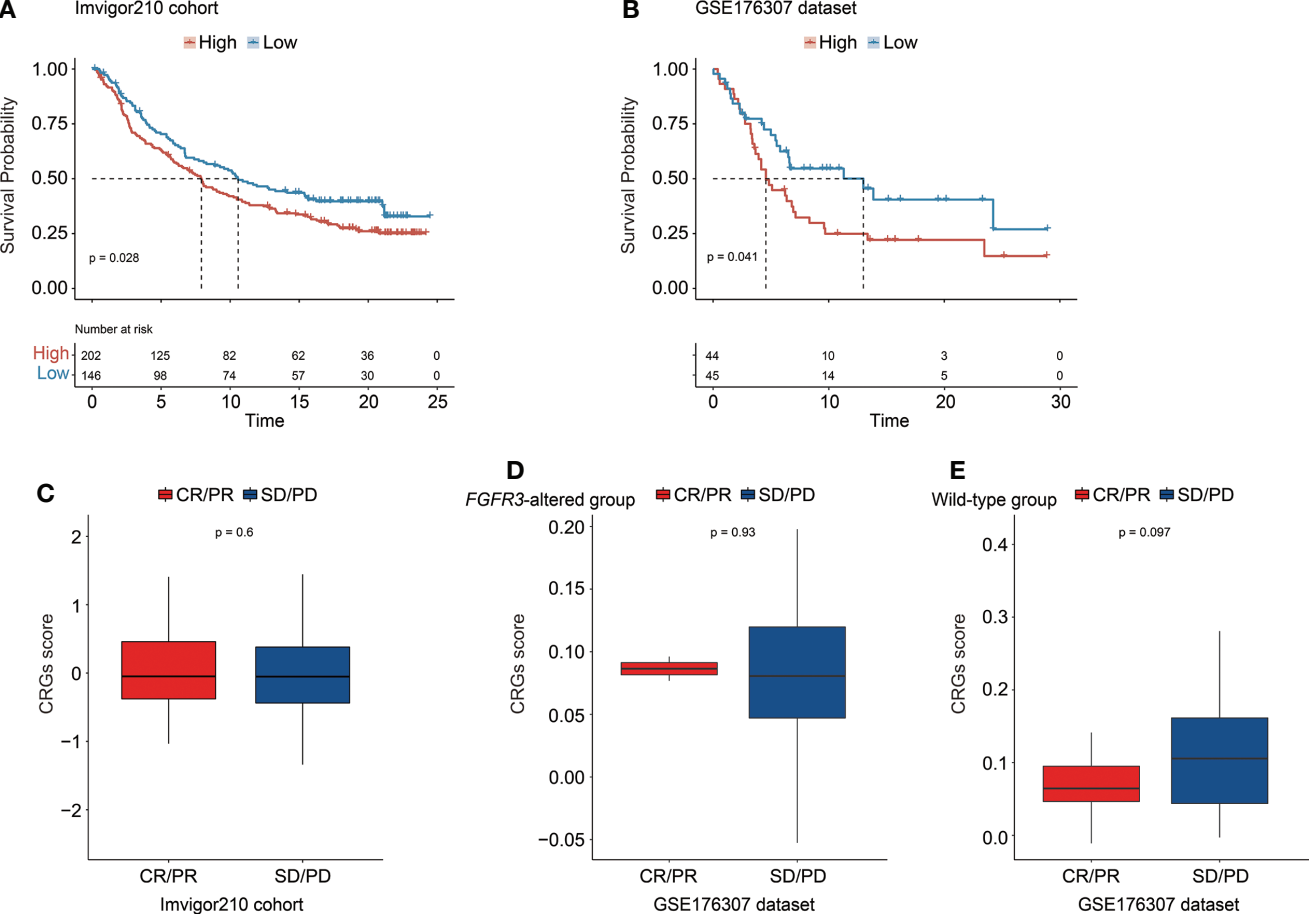
The predictive role of the CRG signature for the response of immunotherapy treatment in the IMvigor210 cohort and the GSE176307 dataset. Kaplan–Meier curve analysis to compare overall survival of patients between high-risk and low-risk groups from the IMvigor210 cohort **(A)** and the GSE176307 dataset **(B)**. Comparison of the CRG score between CR/PR and SD/PD groups in the IMvigor210 cohort **(C)** and in the groups of patients with altered *FGFR3* **(D)** and with wild-type *FGFR3* **(E)** from the GSE176307 dataset.

The CRG signature further exhibited a robust prognosis prediction ability, and CRG score was negatively correlated with the survival of UC patients treated with PD-1/L1 blockades (median OS in the IMvigor210 cohort: 7.92 months vs. 10.58 months, *p* = 0.028, **Figure 12A**; GSE176307 dataset: 4.57 months vs. 13.00 months, *p* = 0.041, **Figure 12B**). When exploring the role of the CRG signature predicting the response of immunotherapeutic treatment in the IMvigor210 cohort, no statistically significant difference in CRG score was observed between patients who responded to PD-1/L1 blockades or not (CR/PR vs. SD/PD patients, **Figure 12C**). Regarding patients with altered *FGFR3* in the GSE176307 dataset, the CRG scores were also equivalent between CR/PR and SD/PD patients (**Figure 12D**). For patients with wild-type *FGFR3* in the GSE176307 dataset, it was notably found that patients who completely/partially responded to immunotherapy have slight lower CRG scores than those with stable/progressive diseases (*p* = 0.097, **Figure 12E**).

## Discussion

Bladder cancer, according to the latest cancer statistics worldwide, remains to be one of the most prevalent cancers, with approximately 550,000 new cases annually (27). Recently, next-generation sequencing technology has led to the significant advances in the research field of bladder cancer (28), discovering a number of genomic, transcriptomic, and proteomic biomarkers for predicting diagnosis or prognosis of patients, as well as promoting clinically favorable targeted therapeutics and effective immune therapies. Furthermore, the newly developed methods have been widely applied to explore the tumor immune microenvironment in various cancers, which expanded the repertoire of precision medicine, especially immunotherapy (29). In the present study, it was the first time to investigate the integrative transcriptional characterization of cell cycle checkpoint genes in BC progression, and eventually a novel CRG signature and CRG-based nomogram were established, with remarkably robust and stable capacity in prognosis prediction. The clinical association analysis underlying the CRG signature further disclosed that the high-risk group had more patients who were female, diagnosed at older age, and with more aggressive diseases. In addition, the molecular characterization, including functional differentiation analysis, genomic alteration profiling, and TME, between different subgroups promoted the potential strategies of precision treatment for BC patients.

The early retrospective study proposed a risk model, namely, CCP score, based on the expression signature of 31 CCP genes, which was created to predict the aggressiveness of prostate cancer (30). Afterwards, this 31-CCP gene expression signature exhibited significant prognostic values in various cancers, such as ductal carcinoma *in situ* of the breast (31), clear cell renal cell carcinoma (32), lung cancer (33), and bladder cancer (34). According to the study of bladder cancer, an optimized 12-CCP signature was established, of which the AUC values in predicting patient progression (non-progressor vs. progressor) were 0.70 in the Lindgren cohort and 0.68 in the CNUH cohort. In contrast, it seemed that the CRG signature might outperform the CCP score in prognosis prediction, with the AUC values at 1, 2, 3, 4, and 5 years for OS all beyond 0.75. Furthermore, recent studies have revealed that the regulation of CCP, such as the initiation of replication, the overall rate of replication, and the recovery and resumption of replication forks, was strictly controlled in cancer cells by checkpoints (35–38). In brief, in cancer cells, cell cycle checkpoints could control CCP and further regulate the cancer cell divisions, unless uncontrolled CCP can drive more mutations and genomic instabilities causing the existence of cell cycle and cell apoptosis.

In past decades, a large body of studies focused on the regulation of CCP in tumor progression (39), in which the important role of checkpoints regulating the entire cell cycle processing was often neglected. In the present study, a total of 52 CRGs in cell cycle were found to be significantly correlated with the clinical outcomes of BC patients; meanwhile, the novel CRG signature was constructed as a prognostic model consisting of further selected 23 CRGs. Among these 23 selected CRGs, the expression of *PPP2CB* has already been proven to be involved in promoting BC cell proliferation and migration (40). The expression of checkpoint CUL4A could mediate the degradation of BECN1 protein to alleviate cell autophagy and enhance the growth of BC (41). The overexpressed *PLK2* was detected in BC, and urinary PLK2 protein level was highly correlated with bladder transitional cell carcinoma (42). The suppressed expression of *THOC1* could mediate BC cell apoptosis (43). Except for the in-depth insight into the biological functions of the above-mentioned four CRGs, 19 other identified CRGs involved in the signature, namely, *ANAPC4*, *MMAB*, *B9D2*, *NDEL1*, *SLC25A15*, *PSMA7*, *PSMB10*, *PSMB5*, *PSMB8*, *RAD9A*, *REXO2*, *ARID3A*, *CHMP4C*, *DDX39B*, *FBXO31*, *FBXO6*, *PRPF19*, *RGCC*, and *TIPIN*, were first identified to play a potential role in BC progression, and their detailed functions remained enigmatic, which merited further experimental verification.

Underlying the CRG signature, correlation analysis revealed that signature-associated genes were mainly responsible for the collagen-containing ECM, and ECM-related and MAPK signaling pathways, indicating that the ECM-specific heterogeneity and related signaling pathways between different risk groups mainly caused the significant difference in OS. It has been revealed that COL1A1 protein was markedly upregulated in MIBC, which could activate the epithelial–mesenchymal transition and TGF-β signaling pathway contributing to the proliferation and invasion of bladder cancer cells (44), whereas the significantly downregulated expression of collagen type IV-α1 and α2 (COL6A1 and COL6A2) was found to promote tumor progression in both the NMIBC and MIBC tissue samples (45). Moreover, the tumor-related macrophages could secrete the type I collagen *via* activating the PI3K/AKT signaling pathway to stimulate the development of bladder cancer; meanwhile, the number of macrophages and the expression of M2 macrophage-associated genes (ARG-1, IL-10, and TGF-β) were remarkably elevated in malignant bladder tumor tissue samples (46). Furthermore, TGF-β could prompt the tumor immune escape and the resistance of immune therapy (47), which has been validated in the IMvigor210 cohort in which BC patients not responding to the therapeutic treatment of anti–PD-L1 agent (Atezolizumab) were highly correlated with the TGF-β signature in fibroblasts and commonly had fibroblast- and collagen-rich peritumoral stroma (48). In addition, a single-cell proteomic analysis further revealed the chaotic tumor-associated collagens in the TME of MIBC (49). Thus, therapeutic treatment targeting collagen-specific ECM and related signaling pathways could be a potential regimen for BC patients.

Genomic alteration analysis revealed that the high-risk group had higher prevalence of tumor suppressor genes *RB1*, *FBXW7*, and *NFE2L2*, consistent with the previous findings that the altered *RB1* (50), *FBXW7* (51), and *NFE2L2* (52) were correlated with tumor progression and worse outcomes of BC patients. However, the potential contribution of frequently altered *ASAP2* and *PCSK6* in the progression of bladder cancer remains unclear, needing to be further investigated. As known, the *FGFR3* alterations were mainly enriched in the luminal bladder cancer correlated with better prognosis; specifically, the low-risk group in the present study was also observed to have more patients with *FGFR3* alterations. More impressively, it was uncovered that there was a significant difference in alteration frequency of only one DDR signaling pathway-related gene—*POLE*—between high-risk and low-risk groups, and Kaplan–Meier analysis revealed that MIBC patients with altered *POLE* exhibited a trend of better OS. Owing to the limited number of MIBC patients presenting *POLE* alterations in the present study, this result should be highlighted and verified in the future.

Taken together, the difference in TME, including tumor purity, T-cell viability, and the proportion of TILs, between high-risk and low-risk groups was further investigated. Macrophage M0 and M2 were consistently enriched in high-risk groups, which also had a lower tumor purity expressed by the higher ESTIMATE score and a higher level of exhausted T cells indicated by the higher TIDE score. Similarly, it has been reported that tumor-associated macrophages could restrain the infiltration level of T cells in tumor tissues (53). In addition, altered *FGFR3* is highly enriched in luminal or its papillary subtypes, which have been characterized by immune-suppressive states (50). Controversially, a retrospective analysis revealed that there was no difference in immune checkpoint blockade response between patients with altered *FGFR3* and those with no altered *FGFR3* (54), and a recent study found the equivalent T-cell receptor diversity between these patients (26). Whether the status of *FGFR3* could influence the treatment response by the immune checkpoint blockade is discussed heatedly. In the present study, it was found that the discrepancy of TME between high-risk and low-risk groups among BC patients with wild-type *FGFR3* was a little small, except that low-risk patients had more activated T cells but a lower proportion of tumor-associated macrophages (especially for macrophage M0 and M2), while compared to the BC patients with wild-type *FGFR3*, high-risk patients with altered *FGFR3* had nearly similar predicted infiltrated immune cell proportions and immune responses, but only the expression levels of *TIM3* and *CD47* were equal, such that these BC patients seemed to be more sensitive to immune checkpoint inhibitors targeting TIM3 and CD47, which needed further analysis. Additionally, the low-risk BC patients with altered *FGFR3* exhibited the highest tumor purity and the most activated T cells, which were associated with better treatment responses of immunotherapies (23). However, in the present study, the expression levels of well-known immune checkpoint genes were found to be extremely low. Thus, in clinic, it was highly recommended that immunohistochemistry staining of immune checkpoint(s) should be detected first during immunotherapeutic treatment.

In addition, the different BC molecular subtypes were identified to have distinctive sensitivity to the chemotherapeutic drugs, such as cisplatin, docetaxel, paclitaxel, methotrexate, and doxorubicin, which were commonly used for BC patients (28). The CRG signature further demonstrated its predictive ability in therapeutic treatment response. Currently, cisplatin has been regarded as a typical chemotherapeutic strategy for UC patients, and it mainly functioned to induce cell cycle arrest and cell apoptosis (55, 56). Furthermore, a previous study has revealed that docetaxel could make antiproliferative and apoptotic effects on bladder cancer cells (57). In the present study, it was found that patients with a higher CRG score, regardless of *FGFR3* status, seem to be more sensitive to cisplatin or docetaxel. Although clinical benefits of paclitaxel were limited owing to patients’ resistance, the failure of first-line combination treatment of cisplatin and gemcitabine for advanced and/or metastatic UC patients provided an opportunity for paclitaxel because of its apoptotic effects (58). Through the evaluation analysis of chemotherapy treatment response, it was recommended that low-risk BC patients with wild-type *FGFR3* might benefit more from paclitaxel. Additionally, the CRG signature also exerted its influence on the patients’ sensitivity to methotrexate, especially suitable for low-risk BC patients with wild-type *FGFR3*; however, its applicability for BC patients in clinic was rare, which needed more verification. Doxorubicin has been proven to disturb the regulation of cell cycle and induce cell death, usually as a vital constituent of combination treatment for MIBC patients (59). Indeed, the alteration frequency of *FGFR3* in MIBC patients was relatively lower, compared with NMIBC patients (60, 61). In the present study, we further found that high-risk BC patients with wild-type *FGFR3* were likely to have higher sensitivity to doxorubicin. Collectively, the checkpoint mechanisms in the regulation of cell cycle greatly influenced the response of chemotherapeutic treatment.

The CRG signature also demonstrated its ability in predicting the response of immunotherapeutic treatment. Of note, the CRG signature exhibited better response prediction capacity for BC patients with wild-type *FGFR3*, of whom those with lower CRG score might be more sensitive to the immunotherapeutic treatment with anti-PD-1 or anti-PD-L1 drugs. Regarding patients with altered *FGFR3*, the CRG signature could not differentiate the potential CR/PR and SD/PD patients. As described before, the alteration frequency of *FGFR3* in low-grade and/or early-stage bladder tumors was evidently higher comparatively, such that the CRG signature could perform better in the response prediction of immunotherapeutic treatment for higher-grade BC patients, especially for those with wild-type *FGFR3*, while only 17 BC patients with altered *FGFR3* included in the GSE176307 dataset received immune therapy treatment hence, the ability of the CRG signature to predict the immunotherapeutic treatment response for BC patients with *FGFR3* alterations remained to be explored.

## Conclusions

The present study comprehensively delineated the integrative transcriptional profiling of cell cycle checkpoint genes in the BC progression. The novel prognostic CRG signature and nomogram exhibited good performance in prognosis prediction for BC patients; furthermore, the molecular characterization underlying the CRG signature provided deep insights into key risk factors leading to the aggressive bladder cancer, promoting the development of precision medicine. The predictive role of the CRG signature in treatment response offered potential precision treatment strategies for different BC molecular subtypes.

## Data availability statement

The original contributions presented in the study are included in the article/**Supplementary Material**. Further inquiries can be directed to the corresponding authors.

## Ethics statement

The studies involving human participants were reviewed and approved by The ethics committee of the Chinese PLA General Hospital (S2019–302-01). The patients/participants provided their written informed consent to participate in this study.

## Author contributions

W-WS, J-ZG, YH, and BY proposed and designed this study. W-WS and J-ZG collected raw data. Y-PL, QS, QX, and B-YQ pre-treated raw data and performed the statistical analyses. W-WS, J-ZG, Y-PL, Z-QM, and BY wrote the original manuscript. YH and BY revised the manuscript. All authors have read and approved this study to be published.

## Funding

This study was supported by the Department of Medical Oncology, Senior Department of Oncology, The Fifth Medical Center of PLA General Hospital.

## Acknowledgments

We are very grateful for the support of The Fifth Medical Center of PLA General Hospital and the great efforts made by all participants. Meanwhile, we appreciate the help offered by the Lifehealthcare Clinical Laboratories, Hangzhou, Zhejiang Province, People’s Republic of China.

## Conflict of interest

The authors declare that the research was conducted in the absence of any commercial or financial relationships that could be construed as a potential conflict of interest.

## Publisher’s note

All claims expressed in this article are solely those of the authors and do not necessarily represent those of their affiliated organizations, or those of the publisher, the editors and the reviewers. Any product that may be evaluated in this article, or claim that may be made by its manufacturer, is not guaranteed or endorsed by the publisher.

## References

[1] Hartwell LH Weinert TA. Checkpoints: Controls that ensure the order of cell cycle events. Science (1989) 246(4930):629–34. doi: 10.1126/science.2683079 2683079

[2] Rubin SM Sage J Skotheim JM. Integrating old and new paradigms of G1/S control. Mol Cell (2020) 80(2):183–92. doi: 10.1016/j.molcel.2020.08.020 PMC7582788 32946743

[3] Saldivar JC Hamperl S Bocek MJ Chung M Bass TE Cisneros-Soberanis F. *et al*: An intrinsic S/G2 checkpoint enforced by ATR. Science (2018) 361(6404):806–10. doi: 10.1126/science.aap9346 PMC6365305 30139873

[4] Löbrich M Jeggo PA. The impact of a negligent G2/M checkpoint on genomic instability and cancer induction. Nat Rev Cancer (2007) 7(11):861–9. doi: 10.1038/nrc2248 17943134

[5] Samuel T Weber HO Funk JO. Linking DNA damage to cell cycle checkpoints. Cell Cycle (2002) 1(3):161–7. doi: 10.4161/cc.1.3.118 12429926

[6] Saldivar JC Cortez D Cimprich KA. The essential kinase ATR: ensuring faithful duplication of a challenging genome. Nat Rev Mol Cell Biol (2017) 18(10):622–36. doi: 10.1038/nrm.2017.67 PMC5796526 28811666

[7] Herlihy AE De Bruin RAM. The role of the transcriptional response to DNA replication stress. Genes (2017) 8(3):92. doi: 10.3390/genes8030092 PMC5368696 28257104

[8] Matthews HK Bertoli C de Bruin RAM. Cell cycle control in cancer. Nat Rev Mol Cell Biol (2022) 23(1):74–88. doi: 10.1038/s41580-021-00404-3 34508254

[9] Putti S Giovinazzo A Merolle M Falchetti ML Pellegrini M. ATM Kinase dead: From ataxia telangiectasia syndrome to cancer. Cancers (2021) 13(21):5498. doi: 10.3390/cancers13215498 34771661 PMC8583659

[10] Schepeler T Lamy P Hvidberg V Laurberg JR Fristrup N Reinert T. A high resolution genomic portrait of bladder cancer: correlation between genomic aberrations and the DNA damage response. Oncogene (2013) 32(31):3577–86. doi: 10.1038/onc.2012.381 22926521

[11] Zhang B Miao T Shen X Bao L Zhang C Yan C. EB Virus-induced ATR activation accelerates nasopharyngeal carcinoma growth *via* M2-type macrophages polarization. Cell Death Dis (2020) 11(9):742. doi: 10.1038/s41419-020-02925-9 32917854 PMC7486933

[12] Quan L Shi J Tian Y Zhang Q Zhang Y Zhang Y. Identification of potential therapeutic targets for melanoma using gene expression analysis. Neoplasma (2015) 62(5):733–9. doi: 10.4149/neo_2015_087 26278148

[13] Yuan W Xie S Wang M Pan S Huang X Xiong M. Bioinformatic analysis of prognostic value of ZW10 interacting protein in lung cancer. OncoTar Ther (2018) 11:1683–95. doi: 10.2147/OTT.S149012 PMC5870638 29615843

[14] Akabane S Oue N Sekino Y Asai R Thang PQ Taniyama D. : KIFC1 regulates ZWINT to promote tumor progression and spheroid formation in colorectal cancer. Pathol Int (2021) 71(7):441–52. doi: 10.1111/pin.13098 33819373

[15] Ying H Xu Z Chen M Zhou S Liang X Cai X. Overexpression of zwint predicts poor prognosis and promotes the proliferation of hepatocellular carcinoma by regulating cell-cycle-related proteins. OncoTar Ther (2018) 11:689–702. doi: 10.2147/OTT.S152138 PMC5800459 29440916

[16] Niehans GA Kratzke RA Froberg MK Aeppli DM Nguyen PL Geradts J. G1 checkpoint protein and p53 abnormalities occur in most invasive transitional cell carcinomas of the urinary bladder. Br J Cancer (1999) 80(8):1175–84. doi: 10.1038/sj.bjc.6990483 PMC2362363 10376969

[17] Doherty SC McKeown SR McKelvey-Martin V Downes CS Atala A Yoo JJ. Cell cycle checkpoint function in bladder cancer. JNCI: J Natl Cancer Inst (2003) 95(24):1859–68. doi: 10.1093/jnci/djg120 14679155

[18] Ye Y Yang H Grossman HB Dinney C Wu X Gu J. Genetic variants in cell cycle control pathway confer susceptibility to bladder cancer. Cancer (2008) 112(11):2467–74. doi: 10.1002/cncr.23472 18361427

[19] Hamid ARAH Ridwan FR Parikesit D Widia F Mochtar CA Umbas R. Meta-analysis of neoadjuvant chemotherapy compared to radical cystectomy alone in improving overall survival of muscle-invasive bladder cancer patients. BMC Urol (2020) 20(1):158. doi: 10.1186/s12894-020-00733-z 33054762 PMC7557048

[20] Kamoun A de Reyniès A Allory Y Sjödahl G Robertson AG Seiler R. A consensus molecular classification of muscle-invasive bladder cancer. Eur Urol (2020) 77(4):420–33. doi: 10.1016/j.eururo.2019.09.006 PMC7690647 31563503

[21] Yoshihara K Shahmoradgoli M Martínez E Vegesna R Kim H Torres-Garcia W. : Inferring tumour purity and stromal and immune cell admixture from expression data. Nat Commun (2013) 4(1):2612. doi: 10.1038/ncomms3612 24113773 PMC3826632

[22] Gentles AJ Newman AM Liu CL Bratman SV Feng W Kim D. The prognostic landscape of genes and infiltrating immune cells across human cancers. Nat Med (2015) 21(8):938–45. doi: 10.1038/nm.3909 PMC4852857 26193342

[23] Jiang P Gu S Pan D Fu J Sahu A Hu X. Signatures of T cell dysfunction and exclusion predict cancer immunotherapy response. Nat Med (2018) 24(10):1550–8. doi: 10.1038/s41591-018-0136-1 PMC6487502 30127393

[24] Kim HR Park HJ Son J Lee JG Chung KY Cho NH. : Tumor microenvironment dictates regulatory T cell phenotype: Upregulated immune checkpoints reinforce suppressive function. J ImmunoTher Cancer (2019) 7(1):339. doi: 10.1186/s40425-019-0785-8 31801611 PMC6894345

[25] Balar A Galsky M Rosenberg J Powles T Petrylak D Bellmunt J. Atezolizumab as first-line treatment in cisplatin-ineligible patients with locally advanced and metastatic urothelial carcinoma: a single-arm, multicentre, phase 2 trial. Lancet (London England) (2017) 389(10064):67–76. doi: 10.1016/S0140-6736(16)32455-2 PMC5568632 27939400

[26] Rose TL Weir WH Mayhew GM Shibata Y Eulitt P Uronis JM. Fibroblast growth factor receptor 3 alterations and response to immune checkpoint inhibition in metastatic urothelial cancer: a real world experience. Br J Cancer (2021) 125(9):1251–60. doi: 10.1038/s41416-021-01488-6 PMC8548561 34294892

[27] Bray F Ferlay J Soerjomataram I Siegel RL Torre LA Jemal A. Global cancer statistics 2018: GLOBOCAN estimates of incidence and mortality worldwide for 36 cancers in 185 countries. CA: A Cancer J Clin (2018) 68(6):394–424. doi: 10.3322/caac.21492 30207593

[28] Tran L Xiao J-F Agarwal N Duex JE Theodorescu D. Advances in bladder cancer biology and therapy. Nat Rev Cancer (2021) 21(2):104–21. doi: 10.1038/s41568-020-00313-1 PMC10112195 33268841

[29] Pardo JC Ruiz de Porras V Plaja A Carrato C Etxaniz O Buisan O. Moving towards personalized medicine in muscle-invasive bladder cancer: Where are we now and where are we going? Int J Mol Sci (2020) 21(17):6271. doi: 10.3390/ijms21176271 PMC7503307 32872531

[30] Cuzick J Swanson G Fisher G Brothman A Berney D Reid J. Prognostic value of an RNA expression signature derived from cell cycle proliferation genes in patients with prostate cancer: a retrospective study. Lancet Oncol (2011) 12(3):245–55. doi: 10.1016/S1470-2045(10)70295-3 PMC3091030 21310658

[31] Lazzeroni M DeCensi A Guerrieri-Gonzaga A Pagan E Bagnardi V Macis D. Prognostic and predictive value of cell cycle progression (CCP) score in ductal carcinoma *in situ* of the breast. Modern Pathol (2020) 33(6):1065–77. doi: 10.1038/s41379-020-0452-0 31925342

[32] Askeland EJ Chehval VA Askeland RW Fosso PG Sangale Z Xu N. Cell cycle progression score predicts metastatic progression of clear cell renal cell carcinoma after resection. Cancer Biomarkers (2015) 15:861–7. doi: 10.3233/CBM-150530 PMC12965479 26406412

[33] Bueno R Hughes E Wagner S Gutin AS Lanchbury JS Zheng Y. Validation of a molecular and pathological model for five-year mortality risk in patients with early stage lung adenocarcinoma. J Thorac Oncol (2015) 10(1):67–73. doi: 10.1097/JTO.0000000000000365 25396679 PMC4272230

[34] Dancik G Theodorescu D. Robust prognostic gene expression signatures in bladder cancer and lung adenocarcinoma depend on cell cycle related genes. PloS One (2014) 9(1):e85249. doi: 10.1371/journal.pone.0085249 24465512 PMC3898982

[35] Dimitrova DS Gilbert DM. Temporally coordinated assembly and disassembly of replication factories in the absence of DNA synthesis. Nat Cell Biol (2000) 2(10):686–94. doi: 10.1038/35036309 PMC1255923 11025658

[36] Feijoo C Hall-Jackson C Wu R Jenkins D Leitch J Gilbert DM. Activation of mammalian Chk1 during DNA replication arrest : a role for Chk1 in the intra-s phase checkpoint monitoring replication origin firing. J Cell Biol (2001) 154(5):913–24. doi: 10.1083/jcb.200104099 PMC1255922 11535615

[37] Trenz K Smith E Smith S Costanzo V. ATM And ATR promote Mre11 dependent restart of collapsed replication forks and prevent accumulation of DNA breaks. EMBO J (2006) 25(8):1764–74. doi: 10.1038/sj.emboj.7601045 PMC1440833 16601701

[38] Yan S Michael WM. TopBP1 and DNA polymerase alpha-mediated recruitment of the 9-1-1 complex to stalled replication forks: Implications for a replication restart-based mechanism for ATR checkpoint activation. Cell Cycle (2009) 8(18):2877–84. doi: 10.4161/cc.8.18.9485 19652550

[39] Otto T Sicinski P. Cell cycle proteins as promising targets in cancer therapy. Nat Rev Cancer (2017) 17(2):93–115. doi: 10.1038/nrc.2016.138 28127048 PMC5345933

[40] Sun S Wang Y Wang J Bi J. Wnt pathway-related three-mRNA clinical outcome signature in bladder urothelial carcinoma: computational biology and experimental analyses. J Trans Med (2021) 19(1):409. doi: 10.1186/s12967-021-03061-4 PMC8477531 34579753

[41] Jin H Ma J Xu J Li H Chang Y Zang N. Oncogenic role of MIR516A in human bladder cancer was mediated by its attenuating PHLPP2 expression and BECN1-dependent autophagy. Autophagy (2021) 17(4):840–54. doi: 10.1080/15548627.2020.1733262 PMC8078721 32116109

[42] Tan L-B Chen K-T Yuan Y-C Liao P-C Guo H-R. Identification of urine PLK2 as a marker of bladder tumors by proteomic analysis. World J Urol (2010) 28(1):117–22. doi: 10.1007/s00345-009-0432-y 19506885

[43] Lin Y Lin C Huang L Chao T Kuo C Hung L. The suppression of thoc1 in cancer cell apoptosis mediated by activated macrophages is nitric oxide-dependent. Biochem Pharmacol (2013) 86(2):242–52. doi: 10.1016/j.bcp.2013.05.009 23688498

[44] Zhu H Chen H Wang J Zhou L Liu S. Collagen stiffness promoted non-muscle-invasive bladder cancer progression to muscle-invasive bladder cancer. OncoTar Ther (2019) 12:3441–57. doi: 10.2147/OTT.S194568 PMC6511250 31123405

[45] Piao X-M Hwang B Jeong P Byun YJ Kang HW Seo SP. Collagen type VI−α1 and 2 repress the proliferation, migration and invasion of bladder cancer cells. Int J Oncol (2021) 59(1):37. doi: 10.3892/ijo.2021.5217 33982770

[46] Qiu S Deng L Liao X Nie L Qi F Jin K. Tumor-associated macrophages promote bladder tumor growth through PI3K/AKT signal induced by collagen. Cancer Sci (2019) 110(7):2110–8. doi: 10.1111/cas.14078 PMC6609800 31120174

[47] Sharma P Hu-Lieskovan S Wargo JA Ribas A. Primary, adaptive, and acquired resistance to cancer immunotherapy. Cell (2017) 168(4):707–23. doi: 10.1016/j.cell.2017.01.017 PMC5391692 28187290

[48] Mariathasan S Turley SJ Nickles D Castiglioni A Yuen K Wang Y. TGFβ attenuates tumour response to PD-L1 blockade by contributing to exclusion of T cells. Nature (2018) 554(7693):544–8. doi: 10.1038/nature25501 PMC6028240 29443960

[49] Feng C Wang X Tao Y Xie Y Lai Z Li Z. Single-cell proteomic analysis dissects the complexity of tumor microenvironment in muscle invasive bladder cancer. Cancers (2021) 13(21):5440. doi: 10.3390/cancers13215440 34771607 PMC8582554

[50] Robertson AG Kim J Al-Ahmadie H Bellmunt J Guo G Cherniack AD. Comprehensive molecular characterization of muscle-invasive bladder cancer. Cell (2017) 171(3):540–556.e525. doi: 10.1016/j.cell.2017.09.007 28988769 PMC5687509

[51] Matumoto T Chen Y Contreras-Sanz A Ikeda K Schulz G Gao J. FBXW7 loss of function contributes to worse overall survival and is associated with accumulation of MYC in muscle invasive bladder cancer. Urologic Oncol: Semin Orig Investigations (2020) 38(12):904–5. doi: 10.1016/j.urolonc.2020.10.048

[52] Choi W Ochoa A McConkey D Aine M Höglund M Kim W. Genetic alterations in the molecular subtypes of bladder cancer: Illustration in the cancer genome atlas dataset. Eur Urol (2017) 72(3):354–65. doi: 10.1016/j.eururo.2017.03.010 PMC5764190 28365159

[53] Joyce JA Fearon DT. T Cell exclusion, immune privilege, and the tumor microenvironment. Science (2015) 348(6230):74–80. doi: 10.1126/science.aaa6204 25838376

[54] Wang L Gong Y Saci A Szabo PM Martini A Necchi A. Fibroblast growth factor receptor 3 alterations and response to PD-1/PD-L1 blockade in patients with metastatic urothelial cancer. Eur Urol (2019) 76(5):599–603. doi: 10.1016/j.eururo.2019.06.025 31272788 PMC6801024

[55] da Silva GN de Castro Marcondes JP de Camargo EA da Silva Passos Júnior GA Sakamoto-Hojo ET Salvadori DMF. Cell cycle arrest and apoptosis in TP53 subtypes of bladder carcinoma cell lines treated with cisplatin and gemcitabine. Exp Biol Med (2010) 235(7):814–24. doi: 10.1258/ebm.2010.009322 20558835

[56] Ho JN Byun SS Lee SE Youn JI Lee S. Multikinase inhibitor motesanib enhances the antitumor effect of cisplatin in cisplatin−resistant human bladder cancer cells *via* apoptosis and the PI3K/Akt pathway. Oncol Rep (2019) 41(4):2482–90. doi: 10.3892/or.2019.7005 30816494

[57] Kassouf W Luongo T Brown G Adam L Dinney C. Schedule dependent efficacy of gefitinib and docetaxel for bladder cancer. J Urol (2006) 176(2):787–92. doi: 10.1016/j.juro.2006.03.072 16813948

[58] Jiménez-Guerrero R Gasca J Flores ML Pérez-Valderrama B Tejera-Parrado C Medina R. Obatoclax and paclitaxel synergistically induce apoptosis and overcome paclitaxel resistance in urothelial cancer cells. Cancers (2018) 10(12):490. doi: 10.3390/cancers10120490 PMC6316685 30563080

[59] Stravopodis D Karkoulis P Konstantakou E Melachroinou S Lampidonis A Anastasiou D. Grade-dependent effects on cell cycle progression and apoptosis in response to doxorubicin in human bladder cancer cell lines. Int J Oncol (2009) 34(1):137–60. doi: 10.3892/ijo_00000137 19082486

[60] Al-Ahmadie HA Iyer G Janakiraman M Lin O Heguy A Tickoo SK. Somatic mutation of fibroblast growth factor receptor-3 (FGFR3) defines a distinct morphological subtype of high-grade urothelial carcinoma. J Pathol (2011) 224(2):270–9. doi: 10.1002/path.2892 PMC3235805 21547910

[61] Knowles MA. FGFR3 – a central player in bladder cancer pathogenesis? Bladder Cancer (2020) 6:403–23. doi: 10.3233/BLC-200373

